# A person-centred and thriving-promoting intervention in nursing homes - study protocol for the U-Age nursing home multi-centre, non-equivalent controlled group before-after trial

**DOI:** 10.1186/s12877-016-0404-1

**Published:** 2017-01-17

**Authors:** David Edvardsson, Karin Sjögren, Qarin Lood, Ådel Bergland, Marit Kirkevold, Per-Olof Sandman

**Affiliations:** 1 The Medical Faculty, Department of Nursing, Umeå University, Vårdvetarhuset, Hus A, plan 5, SE-90187 Umeå, Sweden; 2 College of Science, Health and Engineering, School of Nursing and Midwifery, La Trobe University, Level 4 Austin Tower, PO Box 5555, Heidelberg, 3084 VIC Australia; 3 Lovisenberg Diaconal University College, Lovisenberggt. 15b, 0456 Oslo, Norway; 4 Department of Nursing Science, Faculty of Medicine, Institute of Health and Society, University of Oslo, Postboks 1130, Blindern, 0318 Oslo, Norway; 5 Division of Caring Sciences, Depart Department of Neurobiology, Care Sciences and Society (NVS), Karolinska Institutet, Stockholm, Sweden; 6 Department of Health Sciences, University of Technology, Luleå, Sweden

**Keywords:** Person-centred care, Thriving, Residential aged care, Long-term care, Residents, Relatives, Staff, Job satisfaction, Satisfaction with care, Intervention studies

## Abstract

**Background:**

The literature suggests that person-centred care can contribute to quality of life and wellbeing of nursing home residents, relatives and staff. However, there is sparse research evidence on how person-centred care can be operationalised and implemented in practice, and the extent to which it may promote wellbeing and satisfaction. Therefore, the U-Age nursing home study was initiated to deepen the understanding of how to integrate person-centred care into daily practice and to explore the effects and meanings of this.

**Methods:**

The study aims to evaluate effects and meanings of a person-centred and thriving-promoting intervention in nursing homes through a multi-centre, non-equivalent controlled group before-after trial design. Three nursing homes across three international sites have been allocated to a person-centred and thriving-promoting intervention group, and three nursing homes have been allocated to an inert control group. Staff at intervention sites will participate in a 12-month interactive educational programme that operationalises thriving-promoting and person-centred care three dimensions: 1) Doing a little extra, 2) Developing a caring environment, and 3) Assessing and meeting highly prioritised psychosocial needs. A pedagogical framework will guide the intervention. The primary study endpoints are; residents’ thriving, relatives’ satisfaction with care and staff job satisfaction. Secondary endpoints are; resident, relative and staff experiences of the caring environment, relatives’ experience of visiting their relative and the nursing home, as well as staff stress of conscience and perceived person-centredness of care. Data on study endpoints will be collected pre-intervention, post-intervention, and at a six-month follow up. Interviews will be conducted with relatives and staff to explore experiences and meanings of the intervention.

**Discussion:**

The study is expected to provide evidence that can inform further research, policy and practice development on if and how person-centred care may improve wellbeing, thriving and satisfaction for people who reside in, visit or work in nursing homes. The combination of quantitative and qualitative data will illuminate the operationalisation, effects and meaning of person-centred and thriving-promoting care.

**Trial registration:**

The trial was registered at ClinicalTrials.gov March 19, 2016, identifier NCT02714452.

## Background

Current demographic changes with rapidly ageing populations pose challenges for aged care in terms of innovative, cost-effective and evidence-based ways to support wellbeing and health in frail older people [1, 2]. Aged care has predominantly had a negative societal image in many countries [3], and residents in nursing homes face the risk of limited possibilities to engage in self-selected and valued activities, and to keep in touch with the community outside of the nursing home [4, 5]. This involves serious threats to experiences of health and thriving for residents, whilst relatives report barriers to interacting with and being involved in care in the nursing home to the extent they wish [6, 7]. In addition, a negative societal image of aged care has implications for nursing home staff job satisfaction, contributing to challenges with recruiting and retaining skilled care professionals [3]. Previous research has also shown that nursing home staff experience high levels of stress [8]. Ideally, nursing homes places where frail older people can be supported to thrive despite illness and frailty [9, 10], but in reality many nursing homes struggle to provide more than just the basic physical care. Is it more surviving than thriving?

Thriving is an emerging concept in long-term care, referring to a person’s subjective experience of wellbeing in relation to the place in which they live. From the perspective of frail older people, thriving has been described as experiences of a good life, despite ill health and dependence [11–13]. An increasingly recognised facilitator of thriving within institutional environments is a caring environment, which refers to a holistic experience of the interaction between the physical and psychosocial environment, people’s doing and being within the environment, and a shared philosophy of care [14]. Person-centred care has been described as a philosophy of care to promote independence, authority and choice, and shared decision making for nursing home residents and their relatives [15–17]. However, there is an ongoing discussion in the literature on how person-centred care can be operationalised and implemented in practice, and the extent to which this actually can promote thriving, wellbeing and satisfaction for nursing home residents, relatives and staff. As described by Brownie [18], the concept and philosophy of person-centred care has been well described, developed and refined through conceptual work, and a number of studies have explored a variety of interventions with different endpoints [18]. Some interventions have been related to specific care situations such as bathing, mouth care or morning care [19–21], and others have focused on developing the physical environment to meet the needs of people with dementia [22, 23]. A few intervention studies have had a multi-dimensional, holistic, and individually tailored approach [24, 25], or been part of the conceptual development of person-centred care for example through life story work [26, 27]. Yet, conclusive evidence for effects on resident wellbeing and satisfaction has not yet been clearly established [22, 24, 25, 28, 29]. In addition, even though there is some emerging evidence for positive effects on job satisfaction and work related stress from providing person-centred care among care staff [30, 31], and higher satisfaction with care among relatives [32], further exploration has been recommended [33].

To conclude, the literature indicates that interventions aiming to improve the situation of nursing home residents, relatives and staff would benefit from having a person-centred approach [33, 34], even if further evidence is needed [18]. No previous controlled trials have been located that have evaluated effects and meanings of a person-centred and thriving promoting intervention for nursing home care on resident, relatives’ and staff endpoints. This protocol aims to fill this gap in knowledge [35].

### Theoretical framework

The theoretical framework for the intervention is based on the concepts of *person-centredness*/*person-centred care, thriving* and *caring environments.*

#### Person-centredness/person-centred care

Person-centredness is described as an ethical approach to human interaction, grounded in a fundamental understanding of humans as autonomous reflective persons with capacity and freedom to be the person that they want to be [36]. Implying recognition, respect and trust, person-centredness honours the absolute freedom, value and decision making capacity of each person, regardless of age and physical and cognitive functioning, and emphasises that personhood and dignity needs protection in severe frailty [16]. The ethics of person-centredness is increasingly being operationalised into person-centred care in clinical settings by taking the perspective of the person in need of care as well as the family members as constant point of reference, facilitating shared decision-making, personal authority and choice [15, 16, 37, 38]. In relation to care staff, person-centredredness involves valuing them as individuals by recognising and allowing for the person behind the role to emerge and to draw upon the unique contribution their personalities, experiences, skills and histories can bring to the work [39]. Some models exists that conceptualises the concept of person-centred care, for example Dementia Care Mapping (DCM), the VIPS framework [24, 25], and the Person-Centred Nursing framework (PCN) [40]. These models conceptualises person-centred care as working actively with the older person’s lifestory, personalising care and environment, establishing partnerships and shared decision-making, and prioritising social relations and interactions through the involvement of relatives in the care process.

#### Thriving

Thriving refers to a person’s well-being in relation to the place of stay, and can be understood as a result of an effective mobilisation of both individual and social resources [41], with the aim to support older people’s possibilities to adjust well into their current life situation and the place of stay [42]. As described by Bergland & Kirkevold [42], thriving involves two core and five additional dimensions. The core dimensions represent resident attitudes towards living in a nursing home, and the quality of care and caregivers. The additional dimensions involve qualities in the physical environment, positive relationships with other residents, participation in meaningful activities, opportunities to go outdoors, and relationships with family [42]. Thus, experiences of thriving seem to depend upon how people perceive their present situation in relation to different physical and psychosocial environment factors, i.e. the person-environment interaction and fit [13, 42].

#### Caring environment

The concept of caring environment refers to experiences of an institutions physical and psychosocial environment and the extent to which this is perceived to be caring. Caring environments have been described as emerging from an interaction between aesthetic features of the physical environment and the qualities of people’s doing and being in the environment, influenced by an explicit or implicit philosophy of care [14]. Caring environments have been recognised as influencing satisfaction and wellbeing for nursing home residents, relatives and staff [14], and there is an abundance of previous research showing that the physical environment of care can facilitate or obstruct independence, positive interactions, and meaningful participation in activities [15, 22, 43, 44]. Environments containing objects and symbols that are aesthetically appealing and familiar has also been shown to facilitate positive distractions, and contribute to perceptions of high quality care and wellbeing [14]. Moreover, an aesthetically pleasing environment can be perceived as a way of valuing the life, work and visits of people within nursing home settings [14]. An essential aspect of care staff’s contribution to the caring environment is a willingness to serve in their doing and being, and to do something more than the obvious and expected for residents and their relatives to support positive experiences [14]. Expressions of a willingness to serve have been connected to increased satisfaction with care for residents and relatives [14, 45, 46].

## Methods

The overall aim of the U-Age nursing home study is to evaluate effects and meanings of a person-centred and thriving-promoting intervention in nursing homes. More specifically, the following research questions will be explored:

1. Will the intervention increase resident’s thriving (primary endpoint), and positively affect their experience of the care environment (secondary endpoint)?
2. Will the intervention increase relatives’ satisfaction with care (primary endpoint), enhance their experience of visiting their relative and the nursing home, and positively affect their experience of the care environment (secondary endpoints)?
3. Will the intervention increase staff’s job satisfaction (primary endpoint), decrease their stress of conscience, increase the perceived level of person-centredness of care and positively affect their experience of the care environment (secondary endpoints)?
4. What are the experiences and meanings of the intervention as described by relatives and staff?

The study is designed as a multi-centre, non-equivalent controlled group before-after study with participating sites in Victoria (Australia), Oslo (Norway) and Västerbotten (Sweden). Two nursing homes at each site have been allocated to either intervention or control, in total three intervention and three control facilities across the three international sites. Study specific surveys will be used to collect data on primary and secondary endpoints, with study endpoints being analysed post-intervention, and at six-month follow-up. Qualitative research interviews will be conducted post-intervention to illuminate experiences and meanings of the intervention as described by relatives and staff.

### The intervention

The intervention will be conducted over 12 months and consists of monthly one-hour workshops and reflective evaluation activities between workshops. Inspired by the process and evidence from a previous complex nursing home intervention [35] this intervention will be based on an interactive step-wise pedagogical framework consisting of a) knowledge translation, b) knowledge generation, and c) knowledge dissemination (Table 1).

**Table 1:** Overview of the theoretical and pedagogical framework, key dimensions and activities of the intervention

Theoretical framework	Key intervention dimensions
*Person-centredness/person-centred care* *Caring environment* *Thriving*	1. Doing a little extra 2. Developing a caring physical environment 3. Assessing and meeting highly prioritised psychosocial needs
Pedagogical framework	Key intervention activities
*Knowledge translation*	1. One introductory two-hour lecture on the intervention theory and pedagogy for all direct care staff at the three intervention sites.
*Knowledge generation*	2. Monthly reflective, interactive workshops focusing on attaining, analysing, reflecting and discussing evidence in relation to practice. Staff will conduct evaluation tasks between workshops to critically analyse their current routines, environment and care provision in relation to the three intervention dimensions.
*Knowledge dissemination*	3. Staff will report and discuss the knowledge gained from their evaluation task at local site-specific workshops. Staff will also report and discuss the knowledge gained from the intervention at two international knowledge-sharing seminars, one midterm and end-term.

#### Knowledge translation

During the first step, one introductory two-hour lecture will be provided to all direct care staff at the three intervention sites. The aim of the lecture will be to introduce the theoretical framework, pedagogical framework and research-based evidence that underpins the intervention.

#### Knowledge generation

Staff will take part in 12 reflective learning workshops. These workshops will move systematically through the phases of: a) presenting and discussing the best available evidence, b) discussing how research evidence can be understood and implemented in daily care c) identifying and planning for site-based reflection and evaluation activities, c) performing the site-based reflection and evaluation activities, d) participating in follow-up discussions, analyses and reflection on all evaluation activities. The workshops will be led, supervised and supported by university staff from the research group (KS, QL, ÅB), who will guide staff through the processes of attaining, analysing, reflecting and discussing evidence and evaluation activities. The evaluation activities will be conducted between workshops, which means that staff will be asked to critically analyse their current practice, routines, environment and care provision in relation to the three intervention dimensions; doing a little extra, developing a caring physical environment, and assessing and meeting each resident’s highly prioritised psychosocial needs. They will then be asked to report back their experiences at the subsequent workshop.

#### Intervention dimensions

1. *Doing a little extra.* Participating staff members will be introduced to evidence and understandings on how to facilitate thriving and wellbeing by embodying a willingness to serve in their everyday doing and being, striving to enhance the experiences for residents, relatives and staff by doing a little extra for others. This can mean bringing an unexpected cup of coffee, taking someone outdoors or reporting the latest results from local sports events. Staff will be asked to reflect over and document the little things that they do in everyday activities, actions and interactions with the aim to do something extra for residents, relatives and colleagues to facilitate positive experiences. Documented actions and interactions will be shared and reflected on in the workshops.
2. *Developing a caring physical environment.* Participating staff members will be introduced to evidence and understandings on how to create an aesthetically appealing physical environment that symbolises quality care and respect for each person’s value and worth. Staff will be introduced to, and discuss, ways in which the physical environment can facilitate positive distractions and community, and facilitate a sense of home and value among residents and relatives. They will also discuss how the physical environment can support relatives’ feeling of being welcomed to the nursing home, as well as expressing and symbolising the value of, and the professionalism expected from, each staff member. Staff will be asked to conduct observations of the physical environment and to interview residents and relatives to explore how the environment of their unit can be improved in order to be experienced as caring. Environmental enrichments in the nursing homes’ common areas, such as shared living rooms, sitting/dining rooms and corridors, will be financially supported by the research group.
3. *Assessing and meeting highly prioritised psychosocial needs.* Participating staff members will be introduced to evidence and understandings on how to assess, acknowledge and meet each resident’s most highly prioritised psychosocial needs. The residents’ life histories will be used as a tool to facilitate the understanding of each person’s needs and how these needs are prioritised by the residents, together with if and how such priorities change. Staff will be asked to interview residents and relatives with a focus on preferences, values and highly prioritised psychosocial needs. Staff will also participate in group discussions and reflections on how to integrate these needs into the everyday care and routines of the unit, and to decide on the implementation of activities to meet those needs. All staff will be asked to document the activities they conducted to meet the most highly prioritised needs of a resident.

#### Knowledge dissemination

The third step consists of reporting back on the evaluation activities within and across the international sites to facilitate knowledge dissemination between participants. These activities will include two site-specific workshop discussions, as well as mid- and end-intervention international knowledge-sharing. All participating staff members will be invited to report the experiences and results of their reflective evaluation activities to their site-specific colleagues, with a focus on sharing knowledge within units and presenting their experiences, reflections and conclusions. Second, across-site knowledge sharing will be employed where each site will present their processes and achievements through webcasts and recorded presentations.

### Control facilities

The staff at the three control facilities will receive the same introductory two-hour lecture on the theoretical and practical foundations of person-centredness, person-centred care, thriving and caring environment as the intervention sites. Following this lecture, they will continue their practice without further involvement from the research team. Once the research project has been completed, the control facility will be provided the intervention protocol and support to facilitate implementation of the intervention.

### Inclusion criteria, recruitment, and study sample

To be included in the study, nursing homes must meet the following inclusion criteria: a) managers expressing a need and willingness to participate and support the intervention, b) having about 50 resident beds, and c) employing at least 50 staff members. The recruitment of eligible nursing homes will be purposively conducted at each international site using the researchers’ clinical networks. Nursing home managers will be given/provided verbal and written information on the eligibility criteria, the purpose of the study, the intervention and its implementation process. Before commencing the study, written information and a formal invitation to participate in the data collection will be given to all staff members at the nursing homes where managers have consented to participate.

Participants will be included continuously during the intervention, with the following inclusion criteria: residents need to have lived in the nursing home for >1 month by the time of data collection; relatives need to visit the nursing home on a regular basis (>1/month); staff need to work in the nursing home on a temporary and or permanent basis (>1 month contract), and have been working >1 month in the nursing home by the time of data collection. The goal will be to include at least 900 participants in the study; 300 residents, 300 relatives, and 300 staff members. All eligible persons will be given written information on the purpose and outline of the study, and will be given the opportunity to ask the researchers questions regarding the study throughout the study period.

### Data collection

Demographic and study endpoint data will be collected using anonymous surveys distributed to participating residents, relatives and staff members at the intervention and control nursing homes at baseline, post-intervention, and at six-month follow-up. Demographic data include participants’ age, gender and first language. For residents, demographic data will also include assessments of their ability to perform personal activities of daily living, measured by The Katz ADL-index [47], cognitive impairment measured by the Gottfries cognitive scale [48], and neuropsychiatric symptoms as measured by the Neuropsychiatric Inventory - Nursing Home version [49]. For staff, demographic data will also include education and employment. For relatives, demographic data will also include their relation to the resident and how often they visit the nursing home.

### Study endpoints

The primary study endpoints are: residents’ thriving as measured by the Thriving of Older People Assessment Scale [11], relatives’ satisfaction with care as measured by the Pyramid questionnaire [50], and staff job satisfaction as measured by the Measure of Job Satisfaction scale [51].

The secondary study endpoints consist of the perceived caring environment as measured by the Person-centred Climate Questionnaire-patient, −family, and -staff versions [52–54]. Relatives’ experiences of visiting the nursing home will be measured by 12 study specific questions (e.g. *I feel welcome when I come to the nursing home; The nursing home’s physical environment is aesthetically pleasing; I have positive experiences of visiting my relative)* with answers on a scale from 0 = Totally disagree to 10 = Totally agree. Staff members’ stress of conscience will be measured by the Stress of conscience questionnaire [55] and perceived levels of person-centred care will be measured by the Person-centred Care Assessment Tool [56] . All endpoint measures have been tested for validity and reliability in relation to the study population and context. Please see Table 2 for an overview of endpoint measures.

**Table 2:** Study endpoints and measurements

Endpoints	Measurements	Study population	Tested for reliability and validity
Primary Endpoints			
Thriving	The Thriving of Older People Assessment Scale (TOPAS)	Residents	Yes^a^
Satisfaction with care	The Pyramid Questionnaire	Relatives	Yes^a^
Job satisfaction	The Measure of Job Satisfaction (MJS)	Staff	Yes^a^
Secondary Endpoints			
Experience of the caring environment	The Person-centred Climate Questionnaire (patient/family/staff version)	Residents, relatives and staff	Yes^a^
Experience of visiting their relative and the care setting	Study specific questions	Relatives	N/A
Stress of conscience	Stress of conscience questionnaire	Staff	Yes
Person-centred care	Person-centred Care Assessment Tool (P-CAT)	Staff	Yes^a^

^a^ in relation to study population and context

### Blinding

Due to the nature of the intervention, blinding to study allocation will not be possible.

### Power calculation

Sample size calculations have been made based on a medium effect size of 0.30, a two-sided significance level of 0.05 and a power of 0.85. Calculations for the primary *resident* endpoint was based on unpublished results of TOPAS mean values of 154 and SD 22, indicating that a sample of 150 residents from each group would be sufficient to detect pre-post intervention differences of 6.2. Calculations of the primary *relative* endpoint was based on a previously reported Pyramid questionnaire mean of 76 and SD 21 for overall satisfaction in similar context [57], which indicates that a sample of 150 relatives from each group would be sufficient to detect significant pre-post intervention mean differences of at least 8.7. Calculations of the primary *staff* endpoint was based on a previously reported MJS mean of 81 and SD of 14 for job satisfaction in similar context [34], which indicates that a sample of 150 staff from each group would be sufficient to detect significant pre-post intervention mean differences of at least 3.4. Therefore, a total of at least 900 participants will be recruited.

### Statistical analyses

Sample characteristics will be explored using descriptive statistics, and standard analyses adjustments will be made to adjust for baseline differences between groups should such exist. In cases of missing data, sensitivity analyses will be performed to compare results with complete case analyses [58], and different options for imputation will be considered and discussed with statisticians before making a final decision. Differences between intervention and controls will be tested with *χ*2 tests for categorical variables, and independent sample *t*-test for continuous variables. General linear mixed models will be used to test effects of the interventions on primary and secondary endpoints. Potential confounders will be included as covariates. *P*-values of *<*0.05 will be regarded as statistically significant and effect sizes of *>*0.3 will be regarded as clinically significant. IBM SPSS Statistics for Windows, version 22.0 (Armonk, NY, USA: IBM Corp. Released 2013) will be used to analyse data.

### Research interviews

Group and individual interviews will be conducted to generate narrative research data for analyses of relatives’ and staff’s experiences of the intervention. Group interviews will consist of four to six participants in each group, and aims to stimulate broad discussion and sharing of experiences across units, which then can be followed up in individual research interviews. All interviews will be conducted with a purposefully selected sample of staff and relatives from the three intervention sites. Participants will be selected based on their willingness and ability to provide rich data. A total of 25 participants from each international site (*N* = 75) will be recruited for participation in group and individual interviews. Field notes, audio-recordings and verbatim transcriptions will be employed to document data and enable analyses and interpretations.

All interview data will be subjected to a phenomenological hermeneutical analysis to interpret for meanings of the lived experiences of the intervention. The analytical approach will follow the steps outlined by Lindseth & Norberg [59], involving a dialectical movement between naïve interpretation, structural analyses and the evolving comprehensive understanding [59].

### Study protocol amendments

All study sites will follow the study protocol. If modifications to the study protocol will be necessary, they will be discussed and agreed upon in consensus between the research group and the local facilities before implementation to protect the integrity of the intervention. All changes will be documented in a memorandum.

### Dissemination policy

The results of the study will be reported in international peer-reviewed journals as well as at national and international conferences, and for relevant society and community stakeholders as appropriate.

## Discussion

This protocol outlines the design and procedure for an international multi-site trial within nursing homes. Factors such as limited research cultures, high turnover of staff and residents, and unpredictable external forces such as regulatory organisational changes, suggest that conducting complex interventions in nursing homes is challenging [60]. Therefore, this study protocol can be regarded an important blueprint for both the conduct and evaluation of the intervention, as well as for the communication of information to stakeholders and/or the scientific community. A number of elements and processes are described based on experiences from interventions carried out in similar contexts, such as the theoretical and pedagogical framework for the intervention [61–64]. Anchoring the intervention in a theoretical framework consisting of established theories and evidence-based interventions, as well as a pedagogical framework that moves between knowledge translation, generation and dissemination is a strength of the study. Additionally, previous experiences from similar implementation protocols [35, 63] can be seen as strengthening the study.

The multisite approach can be a strength as well as a weakness of the study, a strength in the sense that the processes, experiences and results will have international applicability and relevance, and a weakness in the sense that there are dimensions that risks deviating slightly between sites. Thus, balancing contextual relevance with standardised content will be a challenge. To secure that the intervention will be as identical as possible across the three sites, the same detailed education material and facilitators guide will be used across sites, and weekly calibration meetings will be held between the three researchers leading the intervention at each site. The collaboration between the research group and the nursing home management and direct care seems essential for a successful intervention. Therefore, the choice of including nursing homes from the researchers’ existing network is regarded as a strength of the study. However, this also excludes the possibility of other nursing homes in need for improvement to participate. Thus sample representativity can be seen as a weakness of the study.

In terms of the study population, frail older people have often been excluded from research due to their vulnerable position and impaired possibility to provide informed consent. However, as the proportion of frail older people increases, the importance of developing research-based knowledge about good care increases, as well as the need to include residents’ perspectives. A main goal of introducing person-centred care in nursing homes is to shift focus from tasks to the person. This study can be seen to challenge those existing models for nursing home care that focuses mainly on the completion of tasks and/or medical care. This study will instead promote a model that focuses on supporting thriving, satisfaction and general wellbeing in residents as well as in relatives and staff. The study can contribute to the knowledge on how to operationalise and implement person-centred care in nursing home contexts. The results are expected to provide evidence to inform further research, policy and practice development on if and how person-centred and thriving promoting care can improve wellbeing and satisfaction for residents, relatives and staff within nursing homes.

## Abbreviations

DCM: Dementia Care Mapping
MJS: The Measure of Job Satisfaction
P-CAT: Person-centred Care Assessment Tool
PCN: The Person-Centred Nursing framework
TOPAS: The Thriving of Older People Assessment Scale
